# Use of Head-Mounted Inertial Sensors for Mobility Tasks: Protocol for a Scoping Review

**DOI:** 10.2196/67628

**Published:** 2025-12-08

**Authors:** Will Sloan, Grace Elizabeth MacDonald, R Bruce Wallace, Rafik Goubran, Heidi Sveistrup

**Affiliations:** 1 Department of Systems and Computer Engineering Faculty of Engineering Carleton University Ottawa, ON Canada; 2 Bruyère Health Research Institute Ottawa, ON Canada; 3 Faculty of Health Sciences University of Ottawa Ottawa, ON Canada

**Keywords:** inertial sensor, mobility, accelerometer, human activity recognition, inertial measurement unit, sensor, wearable device, scoping review

## Abstract

**Background:**

Human activity recognition (HAR) is the use of technology to detect a person’s movements. Sensors can be passive like cameras monitoring an area, or active like those attached to a person’s watch. HAR is a burgeoning field whose uses span from personal health tracking to at-home physical degradation monitoring. The benefit of having sensors attached to a person for constant HAR tracking can be seen in the personal fitness field where people track their number of steps or distance ran. For older adults, HAR can be used in combination with mobility tasks like sit-to-stand, timed-up-and-go, and other tasks to monitor a person’s ability to be self-sufficient and to live alone at home. Together, HAR and mobility tasks are an effective method to measure health, especially in older adults. Consumer wearables like Fitbits and Apple watches are currently being used to measure many of these mobility tasks. The wide adoption of these devices is a result of their ease of use and simplicity. Despite this, most wearables go unused shortly after purchase. A potential solution is the use of consumer devices that are already adopted like headphones or hearing aids. These devices can perform the same measurements as the wearables but have the advantage of being used for other reasons than health monitoring. Despite this, no scoping review has summarized the current capabilities of what can be done using head-worn sensors.

**Objective:**

We aim to understand how head-worn devices have been used to monitor dynamic mobility tasks and if they have been compared to traditional methods (like smartwatches or in-sole sensors).

**Methods:**

This scoping review will be guided by the PRISMA-ScR (Preferred Reporting Items for Systematic Reviews and Meta-Analyses extension for Scoping Reviews) framework. There is no time limit to when the papers must have been published. The databases included in the search are PubMed, Engineering Village, SCOPUS, and Web of Science. Using pre-established inclusion and exclusion criteria, 2 reviewers will screen each paper. We will follow 3 stages of the screening process: abstract and title screening, full-text screening, and full-text data extraction.

**Results:**

The search will be started once this scoping review protocol is published.

**Conclusions:**

Head-mounted devices have become part of everyday life and many already contain accelerometers or inertial measurement units. This scoping review will examine how these sensors have already been used in research to measure mobility tasks, and determine how consumer devices can be adapted to improve the lives of regular people.

**International Registered Report Identifier (IRRID):**

DERR1-10.2196/67628

## Introduction

### Background

Physical activity is defined as the energy expenditure from bodily movement [1] and has been shown to help prevent heart disease, type 2 diabetes, and many other ailments [2]. Given the health implications, enabling a person to track their own physical activity to help them reach the recommended levels can have positive effects on their health [3]. Similarly, this tracking can also be harnessed by medical professionals to identify where problems may stem from or to monitor a patient’s adherence to a prescribed medical routine. Physical activity is especially important for older adults as a sedentary lifestyle increases the percentage of body fat and decline of lean body mass, causing a positive feedback loop [1]. At-home monitoring of one’s physical activity levels can facilitate preventative action and enable tracking of their progress over time [4]. This technology relies on human activity recognition (HAR)—a field of research focused on detecting what a person is doing by using sensors. Closely tied to physical activity is a person’s mobility level, which is the ability to move independently through the day [5]. Without a certain level of mobility, a person cannot perform physical activity to prevent health degradation. In a clinical setting, a person’s level of mobility can be assessed using mobility tasks like sit-to-stand (STS), timed-up and go (TUG), and six-minute walk tasks. Unlike activity levels, these tasks have normative scores that are used as part of the assessment [6]. This review will include both clinical mobility tasks along with generic physical activity detection.

HAR systems can be implemented using environmental sensors (like cameras) or body-worn devices, each with distinct advantages and limitations. Camera-based systems face challenges with framing, clothing variations, and lighting conditions that can affect algorithm performance [7]. Wearable devices circumvent these issues by directly capturing body movement through inertial measurement units (IMUs), which record tri-axial acceleration and angular velocity data [8]. These sensor data enable extraction of basic activity metrics like step counts [9] and energy expenditure [10], as well as detailed gait parameters including walking speed, step length, cadence, and step symmetry. These gait characteristics have clinical significance, with research showing disease-specific patterns such as prolonged stance times following stroke [11], reduced medial-lateral acceleration after concussion [12], and shuffling gait patterns in Parkinson disease [13]. While walking speed has been used as an effective metric to measure a person’s physical health [14], other movements are used in medical testing. An example of this is a 5 times STS test that is used for determining fall risk in patients with Parkinson disease [15]. Several mobility tests can be performed, like a 5 times STS test, six-minute walk test [16], and the TUG test [17], to name a few. These tests provide data for extracting the gait characteristics mentioned earlier. In this review, we will attempt to include all mobility tests by searching for generic terms like walking, mobility transition, and others, along with some named mobility tests mentioned above.

Despite the many ways people can benefit from using wearables, these devices often see a low adoption rate. Participants in studies investigating why people would abandon their devices often mention that they are not the target users for these devices [18]. Since these devices are purchased with the intent of physical activity tracking [19], the person loses interest in the device if they stop exercising. Even if the person is not exercising, the benefits of monitoring the person’s activity levels do not change. Instead, the wearable device they use for physical activity tracking should have some primary purpose that will cause them to adopt the device in the long term. Examples of such devices are hearing aids and earbuds. Hearing aids are often needed and used by older adults [20] who would benefit from physical activity monitoring. Although there is a large need for using hearing aids, there is still difficulty with their adoption for similar reasons as wearables [21]. Despite this problem, if the older adults who use hearing aids also adopt physical activity monitoring, there would still be a large benefit.

Earbuds are another popular but less suitable option. Since they are not worn the entire day, they will not capture as much data as hearing aids. Unlike hearing aids, many people use earbuds when they exercise, creating an opportunity to capture still important and usable data.

Head-mounted devices provide a unique challenge compared to other wearables. Unlike other locations of the body, the head’s stability is important for movement of the body [22]. Because of this, the acceleration signals are attenuated as they pass through the trunk [23,24]. This causes the head acceleration signals to have a lower frequency [23] than other locations like the trunk. This attenuation process is also not uniform, with some directions having more attenuation than others [23]. Furthermore, there are differences in the effectiveness and method of attenuation as the person ages [25,26] and between genders [27,28]. When moving out of the laboratory into free-living environments, many characteristics in the data can change in unexpected ways. Knowing this, we intend to investigate what can be done using head-worn sensors for gathering data on activity levels and mobility tasks.

### Prior Work

To our knowledge, no scoping review has focused on the use of head-worn IMUs for mobility tasks. The closest is Ionut-Cristian’s [29] scoping review, which focuses on head motions quantified by IMUs. Their review covered all movements with a focus on head gestures and their integration in augmented reality. Their goal was to contextualize IMU technology for helping people with partial or total paralysis. This review will focus on any paper using head-worn IMUs for capturing the previously discussed mobility tasks.

Our motivation stems from our previous work in using IMUs in hearing aids for step counts, TUG, and STS [30,31]. When undertaking that work, there was no clear approach for HAR and mobility task analysis for head-worn sensors. Given the difference in signal information from other body parts, we believe that covering what has been done in this area is important for the field.

### Research Questions

The aim of this scoping review is to summarize previous work investigating the use of head-mounted IMUs for gathering mobility measures. Specifically, we aim to identify (1) the outcome measures that can be assessed with head-mounted sensors, (2) the methods for computing these measures, and (3) what devices are used for data acquisition.

## Methods

### Study Design

A preliminary search was performed for related reviews, from which we found the earlier mentioned review [29]. We then met with an academic librarian to help prepare for undertaking the search. The reporting and writing of the scoping review will be guided by the PRISMA-ScR (Preferred Reporting Items for Systematic Reviews and Meta-Analyses extension for Scoping Reviews) checklist.

### Search Strategy

Based on a meeting with the librarian, we iteratively developed our keywords. We carried out the search based on our initial assumptions and then extracted keywords from the relevant articles. The initial search was conducted within IEEE Xplore. The full list of searched terms is included in search strategy (Multimedia Appendix 1). The search was split into three concepts: (1) the mobility task (eg, gait, walk, STS, and TUG), (2) the mounting location or device used (eg, head-mounted device; earables, mastoid, or AirPods [32]; Google Glasses [33]; eSense [34]; and OmniBuds [35]), and (3) the sensor used (eg, IMU, gyroscope, and accelerometer). Mobility task–related keywords have been worded to capture all possible activities and are not restricted to “known” movement sets. The search query has been worded this way to capture movements that may not be directly related to clinical health tests but are still relevant to this review. The databases included in the completed search term list were PubMed, Engineering Village, SCOPUS, and Web of Science. We did not include IEEE Xplore, as it is included within several of the listed databases.

Throughout the search, we used Boolean operators. An example of a search for Engineering Village is provided in Textbox 1. We used the same search approach for each database with the addition of a NOT operator where appropriate.

**An example of a search on Engineering Village.**

(stair* OR ascend* OR descend*) AND (accelerometer OR gyro*) AND (”hearing aid” AND earable* AND earbud*)

### Inclusion Criteria

Textbox 2 lists the inclusion criteria for identifying articles included in the scoping review. We chose the mobility tasks through a combination of our interest in clinical outcomes, what was found in our preliminary search, and previous work we had undertaken [30,31]. Head-worn devices that used an accelerometer in combination with other sensors like gyroscopes, magnetometers, pressure, etc, were included.

**Inclusion criteria.**

*Population*

- Adults.

*Mobility task*

- Gait, walking, and running.
- Sit-to-stand task, timed-up-and-go task, Berg Balance Test, Six-minute walk task, and the 10-meter walk task.
- Ascending or descending the stairs.
- Human activity recognition or activity classification.
- Mobility tests.
- Balance and stability.

*Mounting location or branded devices*

- Attached to the ear, including earbuds, hearing aids, and earables.
- Secured to the head using bands and/or straps.
- Glasses with sensors attached or embedded.
- AirPods, OmniBuds, eSense, or Google Glasses.

*Device Types*

- Any device that measures inertial movement including accelerometers and inertial measurement units.

*Expected outcome measures*

- Types of movements classified (eg, walking vs running vs standing).
- Gait parameters extracted (eg, speed, cadence, and stride length).
- Step count.
- Mobility task–specific variables (eg, time to complete the sit-to-stand task).

*Limits*

- Language: English

### Exclusion Criteria

Textbox 3 lists the exclusion criteria. We excluded articles that reported on the use of multiple sensors where data recording and analysis included fusion with results from a non–head-mounted sensor. An example is the use of a chest-worn IMU in combination with a head-worn IMU.

**Exclusion criteria.**

*Population*

- Children.
- Animals.
- Simulations.

*Mobility task*

- Gestures like nodding and shaking the head.
- Chewing, speaking, and smiling or other related mouth activities.

*Mounting location*

- Sensor fusion between a head-mounted device and a device at any other location.

### Study Screening and Selection

Using the set-out criteria, 2 authors (WS and GM) will independently screen the identified articles. The online software DistillerSR [36] will be used for all parts of the search, including title/abstract screening, full-text screening, and data extraction. The complete extraction sheet is provided in Multimedia Appendix 2, but it was implemented inside of DistillerSR. Any conflicts will be resolved through discussion between the authors until consensus is reached.

## Results

The data collection process has begun, and we expect the results to be published in spring of 2026.

## Discussion

### Principal Results

Currently, extensive research is being carried out on head-worn sensors for mobility tasks. While there has been successful work on implementing step counting [37-40], gait parameter extraction [41-48], the use of virtual reality headsets [49,50], and hearing aid integration [51], there is no comprehensive synthesis of the current state of the research. Presenting a review of this field would be beneficial to show what algorithms have been implemented and what metrics are being used.

The use of head-worn sensors represents an opportunity to capture users' physical health data through devices already integrated into daily routines. The sensors are embedded in commercial products, making them more likely to be adopted by users. With these sensor data, we can track a person's activity levels for personal use or support clinical assessments of mobility function.

However, placing sensors on the head, as opposed to other locations, presents unique technical challenges compared to traditional wrist-worn devices. Head-mounted IMUs capture attenuated acceleration signals, with the degree of the signal dampening varying across individuals due to differences in body mechanics. For reliable health assessments, we must ensure that mobility parameters can be accurately extracted despite these signal characteristics.

This scoping review aims to summarize current approaches for capturing mobility data from head-worn sensors. We will examine the algorithms used, the types of data collected, and sensor placement considerations to provide a comprehensive understanding of current capabilities and limitations.

### Limitations

There may be limitations to what questions this scoping review can answer. Since the wearable field is so rapid and unstructured, we may miss relevant papers. An example of this is the consequence of using abbreviated product names that mask the device’s purpose, making us rely on the tagging system of the databases we searched.

### Conclusions

Human activity recognition is a burgeoning field with many consumer products and companies focusing entirely on the problem. This scoping review aims to answer questions on how sensors in head-worn devices have been used to solve activity detection for various health purposes and then discuss where future research should be focused.

## Appendix

Multimedia Appendix 1 Search strategy.

Multimedia Appendix 2 Data extraction methods.

## Abbreviations

HAR: human activity recognition
IMU: inertial measurement unit
PRISMA-ScR: Preferred Reporting Items for Systematic Reviews and Meta-Analyses extension for Scoping Reviews
STS: sit-to-stand
TUG: timed-up-and-go

## Data Availability

The extraction table data will be made publicly available upon publication of the scoping review.
