# Supplementary material for: Use of Head-Mounted Inertial Sensors for Mobility Tasks: Protocol for a Scoping Review
Source: JMIR Res Protoc. 2025 Dec 8;14:e67628. doi: 10.2196/67628 (PMC12723364; doi:10.2196/67628)
Supplement: Multimedia Appendix 1 [file resprot_v14i1e67628_app1.docx]

**Search Strategy**

| **Major concepts** | | |
| --- | --- | --- |
| Identify the main concepts of your research topic. | | |
| 1. Movement | 2. Sensor | 3. Mounting |

| **Search Strategy** | | | | |
| --- | --- | --- | --- | --- |
|  | | | | |
|  | **Concept 1 AND Concept 2 AND Concept 3** | | | |
|  | Gait or walking or walk* | IMU OR inertial measurement unit* OR inertial sensor* | Head-mount* OR head-worn* |  |
| **OR** | Sit-to-stand OR “chair transfer” OR “sit up” OR “sit down” OR STS OR TUG OR “timed up and go” | Acceleromet* OR gyro* | earable* OR "hearing aid*" OR ear-worn* OR ear-bud* OR earbud* |  |
| **OR** | Stair* |  | Glasses* OR helmet* OR mastoid* OR forehead* |  |
| **OR** | Human activity recognition OR HAR |  | wearable* AND (head* OR ear* OR glasses*) |  |
| **OR** | "activity of daily living" OR ADL OR "activity daily living" OR "activit* daily living" OR "basic activity daily living" OR *ADL OR "instrumental activity daily living" |  |  |  |
