# Supplementary material for: Use of Head-Mounted Inertial Sensors for Mobility Tasks: Protocol for a Scoping Review
Source: JMIR Res Protoc. 2025 Dec 8;14:e67628. doi: 10.2196/67628 (PMC12723364; doi:10.2196/67628)
Supplement: Multimedia Appendix 2 [file resprot_v14i1e67628_app2.docx]

**Data Extraction Sheet**

# Step 1: Study Selection- Title and Abstract Review

| Review title or ID |  |
| --- | --- |
| Study ID (surname of first author and year first full report of study was published (e.g., Smith 2001)  Add contact details of corresponding authors |  |

# General Information

| Date form completed (dd/mm/yyyy) |  |
| --- | --- |
| Name/ID of person extracting data |  |
| Reference citation |  |
| Publication type  (e.g., full report, abstract, letter) |  |
| Notes: | |
| Abstract | |

Continue to screening on next page.

# Study Eligibility Screening (Title/Abstract)

| Study Characteristics | Eligibility criteria  (Insert inclusion criteria for each characteristic as defined in the Protocol- highlight when necessary) | | Eligibility criteria met ? | | | Notes |
| --- | --- | --- | --- | --- | --- | --- |
|  |  |  | Yes | No | Unclear |  |
| Type of Population | Adult People | |  |  |  |  |
| Type of device | Inertial measurement unit/IMU/Accelerometer/Accelerometer + Gyroscope/ Accelerometer+other | |  |  |  |  |
| Device location | Head-mounted/head-worn/behind ear/forehead/mastoid | |  |  |  |  |
| Mounting type | Glasses/helmet/hearing aids/head band/ tape | |  |  |  |  |
| Target variable | Gait/TUG/STS/pedometry/walk/human activity recognition/stride | |  |  |  |  |
| Language | English | |  |  |  |  |
| Pub Type | Online searchable | |  |  |  |  |
| Main objectives   - Objective: estimate walking speed using accelerometer on glasses. | | | | | | |
| Abstract and title screening  INCLUDE  EXCLUDE  Full Text screening  INCLUDE  EXCLUDE | |  | | | | |
| Reason for exclusion |  | | | | | |
| Notes: | | | | | | |

DO NOT PROCEED TO FULL TEXT REVIEW IF STUDY EXCLUDED FROM REVIEW

# Step 2: Full Text Review – Data Extraction

| **Study information not captured in first selection round (title and abstract)** | |
| --- | --- |
| Sample size |  |
| Study design |  |
| Main Findings   - Validated - Feasibility - Accuracy |  |

| **AIM 1: What data from the device is being used to predict the outcome, and how is it being validated?** | |
| --- | --- |
| What device is being used? |  |
| What sensors are on the device? (accel, gyro, gps, etc) |  |
| What is the sampling rate of the device? |  |
| Where is the device located on the head? |  |
| How is the device mounted? |  |
| If it is done, what is done for alignment/calibration? |  |
| What mobility tasks is being recorded? (ex: walking, sts, stairs) |  |
| Is it classification or regression? (ex: detecting when someone is walking - classification, or using data to get a value like step count - regression)  **For regression, make a copy of table 2 for each task.** |  |

Table 2: movement 1

| Mobility Task |  |
| --- | --- |
| What sensor data is used? (ex: uses accel, but no gyro) |  |
| Is there preprocessing and what is it? |  |
| What features of the data are computed for use by the algorithm? (ex: mean of y-axis of the accelerometer and gyro) |  |
| What algorithm is being used? (ex: KNN, SVM, peaks) |  |
| What is the outcome measure of the algorithm? (ex: time, number of steps, stride size) |  |
| What about the movement is being validated? |  |
| What training/testing of the algorithm approach is used? (Ex: cross-validation) |  |
| Settings which algorithm was tested? (ex: in lab, outside unsupervised) |  |
| What device is being used for the gold standard? |  |
| Where is the gold standard device located? (external if not on person) |  |
| How is the outcome being compared with the gold standard? |  |
| Was data separated by some factor and what was it? (ex: separated based on gait speed) |  |
| Statistical results of study for each separation factor? (ex: accuracy) |  |
| Interpretation of study? (ex: algorithm shows promise compared to current methods) |  |
| Notes and Additional Information: | |

Table 2: movement 2. Ignore if only 1 movement.

| Mobility Task |  |
| --- | --- |
| What sensor data is used? (ex: uses accel, but no gyro) |  |
| Is there preprocessing and what is it? |  |
| What features of the data are computed for use by the algorithm? (ex: mean of y-axis of the accelerometer and gyro) |  |
| What algorithm is being used? (ex: KNN, SVM, peaks) |  |
| What is the outcome measure of the algorithm? (ex: time, number of steps, stride size) |  |
| What about the movement is being validated? |  |
| What training/testing of the algorithm approach is used? (Ex: cross-validation) |  |
| Settings which algorithm was tested? (ex: in lab, outside unsupervised) |  |
| What device is being used for the gold standard? |  |
| Where is the gold standard device located? (external if not on person) |  |
| How is the outcome being compared with the gold standard? |  |
| Was data separated by some factor and what was it? (ex: separated based on gait speed) |  |
| Statistical results of study for each separation factor? (ex: accuracy) |  |
| Interpretation of study? (ex: algorithm shows promise compared to current methods) |  |
| Notes and Additional Information: | |

Table 2: movement 3. Ignore if only 2 movements. Make more if needed.

| Mobility Task |  |
| --- | --- |
| What sensor data is used? (ex: uses accel, but no gyro) |  |
| Is there preprocessing and what is it? |  |
| What features of the data are computed for use by the algorithm? (ex: mean of y-axis of the accelerometer and gyro) |  |
| What algorithm is being used? (ex: KNN, SVM, peaks) |  |
| What is the outcome measure of the algorithm? (ex: time, number of steps, stride size) |  |
| What about the movement is being validated? |  |
| What training/testing of the algorithm approach is used? (Ex: cross-validation) |  |
| Settings which algorithm was tested? (ex: in lab, outside unsupervised) |  |
| What device is being used for the gold standard? |  |
| Where is the gold standard device located? (external if not on person) |  |
| How is the outcome being compared with the gold standard? |  |
| Was data separated by some factor and what was it? (ex: separated based on gait speed) |  |
| Statistical results of study for each separation factor? (ex: accuracy) |  |
| Interpretation of study? (ex: algorithm shows promise compared to current methods) |  |
| Notes and Additional Information: | |
